# The Photoreceptor Components FaWC1 and FaWC2 of *Fusarium asiaticum* Cooperatively Regulate Light Responses but Play Independent Roles in Virulence Expression

**DOI:** 10.3390/microorganisms8030365

**Published:** 2020-03-05

**Authors:** Ying Tang, Pinkuan Zhu, Zhengyu Lu, Yao Qu, Li Huang, Ni Zheng, Yiwen Wang, Haozhen Nie, Yina Jiang, Ling Xu

**Affiliations:** School of Life Sciences, East China Normal University, Shanghai 200241, China

**Keywords:** photobiology, transcription factor, White collar complex, *Fusarium asiaticum*, virulence

## Abstract

*Fusarium asiaticum* belongs to one of the phylogenetical subgroups of the *F. graminearum* species complex and is epidemically predominant in the East Asia area. The life cycle of *F. asiaticum* is significantly regulated by light. In this study, the fungal blue light receptor white collar complex (WCC), including FaWC1 and FaWC2, were characterized in *F. asiaticum*. The knockout mutants Δ*Fawc1* and Δ*Fawc2* were generated by replacing the target genes via homologous recombination events. The two mutants showed similar defects in light-induced carotenoid biosynthesis, UV-C resistance, sexual fruiting body development, and the expression of the light-responsive marker genes, while in contrast, all these light responses were characteristics in wild-type (WT) and their complementation strains, indicating that FaWC1 and FaWC2 are involved in the light sensing of *F. asiaticum*. Unexpectedly, however, the functions of *Fawc1* and *Fawc2* diverged in regulating virulence, as the Δ*Fawc1* was avirulent to the tested host plant materials, but Δ*Fawc2* was equivalent to WT in virulence. Moreover, functional analysis of FaWC1 by partial disruption revealed that its light–oxygen–voltage (LOV) domain was required for light sensing but dispensable for virulence, and its Zinc-finger domain was required for virulence expression but not for light signal transduction. Collectively, these results suggest that the conserved fungal blue light receptor WCC not only endows *F. asiaticum* with light-sensing ability to achieve adaptation to environment, but it also regulates virulence expression by the individual component FaWC1 in a light-independent manner, and the latter function opens a way for investigating the pathogenicity mechanisms of this important crop disease agent.

## 1. Introduction

Fusarium head blight (FHB), which is usually caused by the pathogen *Fusarium graminearum*, is known as a global problem devastating small grain cereal crops [1]. Phylogenetic species recognition has revealed that *F. graminearum* sensu lato comprises at least 15 biogeographically structured and phylogenetically distinct species, all of which are known as the *Fusarium graminearum* species complex, or FGSC [2,3,4,5,6,7]. Among the FGSC, *Fusarium asiaticum* belongs to one sub-lineage and is the predominant FHB agent species in East Asia [8]; it is especially prevalent in the wheat production zones of the Yangtze-Huaihe valley in China [9]. Besides being commonly associated with FHB, *F. asiaticum* has also been found to cause postharvest rot on asparagus spears and produce 3A-DON mycotoxin during host infection [10].

Usually, introgression of the disease resistance genes identified in natural sources into elite cultivars represents a reliable route for plant disease management [11]. However, plant sources for FHB resistance are unfortunately limited, and no fully resistant cultivars are yet available [12]. Controlling FGSC-caused diseases will benefit from an in-depth understanding of how the pathogens infect and spread inside the host. The availability of the *F. graminearum* genome [1] has greatly stimulated the research activity on identification of functional genes as well as pathogenicity factors of this phytopathogen [13,14,15]. However, the molecular mechanism of development and virulence regulation is less known in *F. asiaticum* than in *F. graminearum*, although there is an ongoing trend in which *F. asiaticum* becomes more aggressive and devastating than *F. graminearum* in the East Asian area [6,14,16].

Light is an important environmental factor that can extensively influence varied aspects of most living organisms on earth [17]. Filamentous fungi can use light as a general signal for regulating development, metabolism, sexual or asexual reproduction, and other life processes to adapt to a specific ecological niche [18,19,20]. At the molecular level, light is sensed by fungal photoreceptors, leading to activating or suppressing the transcription of photoresponsive genes, which are furthermore considered to result in the accumulation of light-sensing responses.

Light signaling is most extensively studied in the model species *Neurospora crassa*, in which carotenoid biosynthesis and morphological development, including the formation of asexual spores and protoperithecia, are notably regulated by blue light [21,22,23]. The analysis of blind mutants revealed that the white-collar 1 protein (WC-1), a transcription regulator which contains a light–oxygen–voltage (LOV) domain to bind the flavin chromophore, and the WC-2, a second transcription regulator without a chromophore-binding domain, can form a heterodimer called white-collar complex (WCC) to positively regulate the light-induced genes. Besides *N. crassa*, the molecular components for blue light sensing appeared to be widely conserved in the fungal genomes of Ascomycetes, Mucoromycetes, and Basidiomycetes. Moreover, genes under the control of the WCC can be either light responsive or not light responsive, and WC-1 and WC-2 can also have individual functions besides acting cooperatively as the WCC [24,25].

Many fungal species are causing detrimental diseases to mammals and plants, since the outcomes of all the epidemic diseases on earth can be determined by the triangular interactions among host–pathogen–environment [26]. Whether light signaling in fungi is involved in determining the disease outcomes has attracted considerable research attempts to characterize the photoreceptor functions in pathogenic fungi. However, the WCC regulatory circuit demonstrates functional variation among different species of fungal pathogens, and the significance of fungal light-sensing capacity for virulence expression is concluded on a case-by-case basis [27,28,29,30]. Moreover, despite these functional studies of the WCC orthologs in varied phytopathogenic fungi, the following questions remain mysterious: whether sensing light (or the absence of light) by phytopathogenic fungi is essential for pathogenicity, and how to exclude light-independent functions of WCC orthologs when evaluating their contribution in determining fungal pathogenicity.

In this paper, the cloning and characterization of the *Fawc1* and *Fawc2* genes in *F. asiaticum* demonstrate that both *Fawc1* and *Fawc2* are involved in light sensing and regulating pleiotropic fungal development processes, suggesting that FaWC1 and FaWC2 function cooperatively as the WCC to fulfill the photo receptor tasks. However, it is *Fawc1* but not *Fawc2* that is required for virulence. Functional domain analysis of FaWC1 reveals that the LOV and Zinc-finger domains are independently required for light sensing and virulence, respectively. These findings not only expand the knowledge of fungal photobiology but also provide novel insights about the mechanisms for diverged functions of WCC components in determining fungal virulence in a light-independent manner.

## 2. Materials and Methods

### 2.1. Fungal Strains and Culture Conditions

The wild-type strain EXAP-08 was previously isolated from postharvest asparagus spears with serious rot symptoms and characterized as *F. asiaticum*, and it is used as the recipient strain for genetic modifications in this study. The wild-type strain and the resulting mutants are listed in Table 1, and all the fungal strains were purified by single spore isolation and were stored as spore suspension in 20% glycerol at −80 °C. The fungal cultures were grown on complete medium (CM) (10 g glucose, 2 g peptone, 1 g yeast extract, 1 g casamino acids, nitrate salts, trace elements, 0.01% of vitamins, 10 g agar and 1 L water, pH 6.5) for mycelial growth, carboxymethyl cellulose (CMC) medium [31] for conidiation, and carrot agar medium [32] for sexual development. For light-responsive phenotypic analyses, plates were incubated for 3–7 days at 25 °C as indicated, either under illumination (20 W m^−2^ white light obtained with fluorescent bulbs) or under dark conditions.

### 2.2. Sequence Analysis of Fawc1 and Fawc2 of F. Asiaticum

The amino acid sequences of WC-1and WC-2 of *Neurospora crassa* were used to blast against the *Fusarium graminearum* genome at the Ensembl Fungi database (http://fungi.ensembl.org/index.html). The obtained target sequences were used as references to design primers to amplify the corresponding orthologs in *F. asiaticum* wild-type strain EXAP-08. The amplified products were subcloned to T-vector and subsequently sequenced. The obtained sequences of *Fawc1* and *Fawc2* were further aligned with their orthologs from other fungal species retrieved from publically available databases by DNAMAN version 5.2.2 (LynnonBiosoft Company, Pointe-Claire, QC, Canada).

### 2.3. Generation of Mutants and Complementation Strains in F. asiaticum

The flanks 5′ and 3′ of *Fawc1* and *Fawc2* were amplified from EXAP-08 genomic DNA, and the *hph* was amplified from plasmid *p22*. Overlap PCR was performed to obtain the 5′-*Fawc1*-*hph*-*Fawc1*-3′ gene knockout cassettes by mixing an equimolar ratio of 5′-*Fawc1*, *hph*, and *Fawc1*-3′ as templates, and the 5′-*Fawc2*-*hph*-*Fawc2*-3′ cassette was prepared in a similar way. The resulted products were separated by gel electrophoresis and then recovered by a gel extraction kit. The purified gene knockout cassettes (2 µg for each cassette) were subjected to protoplast transformation of the wild-type strain EXAP-08 according to the reported method [33]. To screen for the correct mutants of ∆*Fawc1* and ∆*Fawc2*, the transformants grown on selective media containing 75 µg/mL hygromycin B were purified and subjected to genomic DNA extraction via the standard CTAB protocol. Site specific primers were used to carry out PCR assay to screen for the knockout mutants.

To generate the complementation strains, wild-type *Fawc1* and *Fawc2* connected with their 1.5 kb 5′- and 1 kb 3′-flanking sequences were amplified and subsequently cloned into the flu6 plasmid that contains the geneticin (G418) resistance gene, resulting in *flu6*-*Fawc1*-com and *flu6*-*Fawc2*-com expression vectors. Then, the complementation vectors were transformed into the protoplasts of the corresponding gene deletion mutants. CM containing 100 µg/mL of G418 was used to select the successful transformants. To generate mutant strains carrying truncated FaWC1 that lacked either LOV or ZnF domains, the *Fawc1^∆^*^LOV^ and *Fawc1^∆^*^ZnF^ fragments with deletion of the coding regions for LOV and ZnF domains, respectively, were generated by overlap PCR, and the resulted products were gel-purified and cloned into the flu6 plasmid, leading to the expression vectors *flu6*-*Fawc1^∆^*^LOV^ and *flu6*-*Fawc1^∆^*^ZnF^, which were delivered into ∆*Fawc1* mutant to generate the ∆*Fawc1-C^∆^*^LOV^ and ∆*Fawc1-C^∆^*^ZnF^ strains. All the primers used in the mutant generation and diagnosis PCR reactions are listed in supplementary Table S1.

### 2.4. Extraction of RNA and Quantitative RT-PCR Analysis

For gene expression analysis, samples were prepared as follows: aliquots of 200 μL conidia suspension (10^6^/mL) were inoculated on solid CM medium with cellophane overlays and incubated at 23 °C. The mycelium samples were harvested after either 48 h culture in total darkness, or by ending the 48 h culture with light illumination of indicated time duration. Total RNA was extracted using QIAGEN Reagent (Germany), and 1 μg of each RNA sample was used for reverse transcription with the Prime Script™ RT reagent Kit (Perfect Real Time) (TakaRa Biotechnology, Co., Dalian, China). The real-time PCR amplifications were conducted in a CFX96^TM^ Real-Time System (BIO-BAD, Inc., USA) using TakaRa SYBR Premix Ex Taq (TakaRa Biotechnology, Co., Dalian, China). For each sample, the expression of the β-tubulin gene was used as an internal reference. There were three replicates for each sample. The experiment was repeated three times. The gene expression levels were calculated using the 2^−∆∆Ct^ method.

### 2.5. Growth and Development Phenotyping

Phenotypic differences between the mutant strains and wild type were analyzed by culturing them on CM agar medium in dark and light conditions. Colony morphology was recorded by photographs at four days after inoculation. To assess the self-fertility of each strain, mycelium plugs of 5 mm diameter were excised from the edge of 4-day-old colonies and then inoculated on carrot agar medium. The Petri dishes were put under constant light for four days when the mycelia overgrew the petri dish; then, we added 1 mL of Tween-20 in the petri dish and overwhelmed the aerial mycelia by the back of a spoon. Afterwards, the plates were transferred to near-UV light for homothallic sexual development induction. After two months, the plates were checked for perithecium formation and maturation and photo recorded via a stereomicroscope equipped with a CCD digital camera (SMC168, Moticam Pro 205B Motic, Xiamen, China). For ascospore observation, a very small brick of perithecium was picked off, pressed on a glass slide under a cover glass, and then observed using a microscope (AE30, Motic, Xiamen, China).

### 2.6. Carotenoid Measurement Assay

Mycelium samples grown in liquid CM medium (23 °C, 150 rpm) for three days were collected for lyophilization. For each strain, 200 mg of lyophilized mycelial biomass was added in 750 µL of hexane and 500 µL of methyl alcohol; then, it was homogenized in a Tissuelyser-24 with stainless-steel beads (Jingxin, Ltd., Shanghai, China). The resulted suspensions were centrifuged at 12,000 rpm for 10 min. The supernatant was used to determine the absorbance value (A) at 445 nm. Carotenoid contents of the samples were quantified via the equation: X(mg/100 g) = $\frac{A\;\times \;y\left(\mathrm{mL}\right)\;\times \;1000}{\mathrm{Af}\;\times \;g}$, in which “y”, “Af”, and “g” represent the volume of the extraction buffer, average absorption coefficient of the carotenoid molecular (2500), and the weight of the sample, respectively.

### 2.7. UV Sensitivity Assay

Five microliters of serial diluted conidial suspensions (10^6^, 10^5^, 10^4^ conidia/mL) from cultures grown in liquid straw media was point-inoculated onto CM agar medium. The plates were exposed to UV-C light (provided by UVB HL-2000 Hybridizer, peaking at 245 nm, 0.2 kJ/m^2^) and then allowed to recover in dark or under white light for two days at 23 °C. The UV sensitivity of each strain was determined by comparing the survival of colonial cultures grown in light versus dark after UV exposure.

### 2.8. Virulence Assay

Fungal conidia were harvested from CMC medium and were suspended in liquid minimum Gamborg B5 medium (or GB5, Coolaber, China, containing 0.6 g·L^−1^ GB5 salts, 10 mM glucose, pH 7.0), and the concentration was adjusted to 1 × 10^6^ conidia/mL. The infection assay was conducted according to the reported method [14] with modifications as follows: the wheat (*Triticum aestivum* cultivars Zhongyuan 98–68) coleoptiles grown for two days were cut with a scissor and inoculated with 5 µL conidial suspension, and then they were kept in a transparent box at 23 °C under a 12 h/12 h light cycle and humid condition. The infected samples were photographed every day, and the lesion areas were calculated using the ImageJ software.

### 2.9. Statistical Analysis

The data obtained in this study were analyzed with ANOVA followed by Duncan’s multiple range tests (*p* < 0.05) for means comparison with the use of SPSS 17.0.

## 3. Results

### 3.1. The Orthologs of WC-1 and WC-2 in F. asiaticum

**Figure 1:**
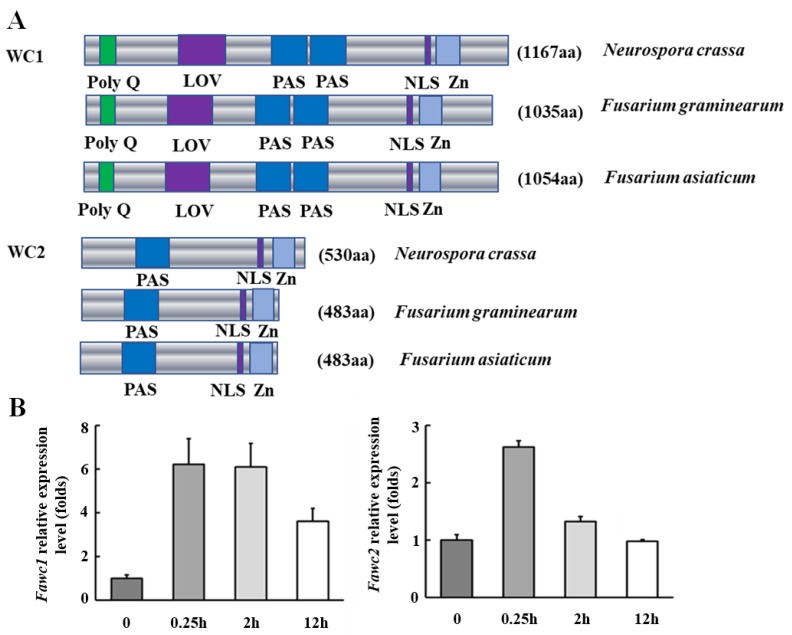
Identification of two photoreceptor genes, *Fawc1* and *Fawc2*, in *F. asiaticum*. (**A**). Schematic demonstration of the domains of WC1 and WC2 orthologs from *N. crassa*, *F. graminearum*, and *F. asiaticum*. Accessions of the amino acid sequences are as follows: WC1 (NCU02356); WC2 (NCU00902); FgWC1 (FGSG_07941); FgWC2 (FGSG_00710); FaWC1 (KX905081.1); FaWC2 (MT019868). Domains of these photoreceptor proteins were analyzed via the SMART online tool (http://smart.embl-heidelberg.de/). PAS: Per-period circadian protein; Arnt: Ah receptor nuclear translocator protein; Sim: Single-minded protein, NLS: Nuclear Location Singal, Zn: Zinc finger binding to DNA consensus sequence. (**B**). Transcript levels of *Fawc1* and *Fawc2* are regulated by light. The horizontal axis indicates light treatment time. The bars present mean values ± SD of three replicate samples.

Since *F. asiaticum* belongs to a sub-lineage of the *Fusarium graminearum* species complex, the orthologous genes of *wc1* and *wc2* in *F. asiaticum*, namely *Fawc1* and *Fawc2*, were identified as follows: BLASTP search of the *F. gaminearum* genome in Ensembl Fungi database, using the amino acid sequences of *Neurospora crassa* WC-1 (NCU02356) and WC-2 (NCU00902) proteins [34] as queries, resulted in the corresponding orthologs namely FgWC1 (FGSG_07941) and FgWC2 (FGSG_00710), respectively. The corresponding genes and their flanking sequences were used as references to design specific primers to amplify the open reading frame (ORF) sequences of their orthologs, *Fawc1* and *Fawc2*, in *F. asiaticum* strain EXAP-08 via the *Pfu* DNA polymerase. Sequencing of the cloned PCR products indicated that *Fawc1* (KX905081.1) and *Fawc2* (MT019868) of *F. asiaticum* showed high similarity to *Fgwc1* (99.05%) and *Fgwc2* (99.39%) of *F. graminearum*. The deduced amino acid sequences of FaWC1 and FaWC2 were analyzed via the SMART online tool, and the results indicated that FaWC1 possessed a Zn-finger DNA-binding domain as well as three PAS domains, of which the N-terminal most PAS domain should be the special subclass called the LOV domain (for light, oxygen, and voltage) being responsible for binding the chromophore molecule of flavin adenine dinucleotide (FAD), while the FaWC2 just essentially contained a single PAS domain and a Zn-finger DNA binding domain. In general, FaWC1 and FaWC2 show high similarity with their orthologs in *F. graminearum* and *N. crassa* (Figure 1A). Gene expression analysis showed that both *Fawc1* and *Fawc2* were induced to peak levels by 15 min light exposure, while the long period (12 h) of illumination caused less induction of the transcription levels of these two genes (Figure 1B).

### 3.2. Generation and Characterization of the ∆Fawc1 and ∆Fawc2 Mutants

**Figure 2:**
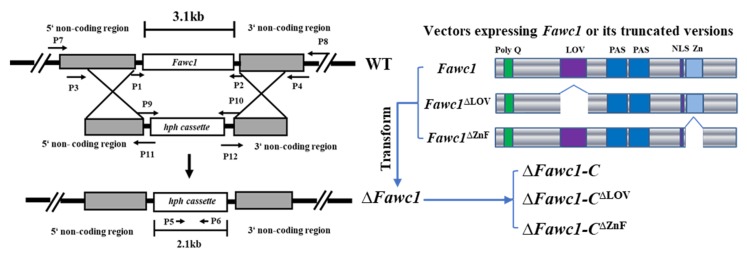
Generation of the transgenic mutants of *F. asiaticum.* Schematic diagrams of homologous recombination occurred between the replacement vector carrying the hygromycin resistance marker (*hph*) and the target gene (*Fawc1*) of *F. asiaticum* strain EXAP-08, resulting in the knockout mutant (∆*Fawc1*). Meanwhile, the wild-type *Fawc1* or its truncated versions, *Fawc1*^∆LOV^ and *Fawc1*^∆Zn^, were amplified from genomic DNA of EXAP-08 and cloned into the flu6 plasmid, and then transformed into the ∆*Fawc1* mutant, resulting in the complementation strain ∆*Fawc1*-*C*, and ∆*Fawc1-C*^∆LOV^ and ∆*Fawc1-C*^∆ZnF^ mutant strains. The knockout mutant ∆*Fawc2* and its complementation strain ∆*Fawc1*-*C* were generated via a similar strategy.

To reveal the functions of *Fawc1* and *Fawc2*, homologous recombination cassettes used for gene knockout purposes were created as shown in Figure 2. After protoplast transformation, the transformants with hygromycin resistance successfully grew up on the selection medium. To characterize the knockout mutants of ∆*Fawc1* and ∆*Fawc2* in which the hygromycin resistance cassette (*hph*) had correctly replaced each target gene, PCR assays with the specific primer pairs were performed with the genomic DNA, and the correct transformants for each gene were selected for further analysis. To verify that the *Fawc1* or *Fawc2* was completely knocked out, reverse transcription (RT)-PCR was applied, revealing that the transcripts of *Fawc1* and *Fawc2* were present in the EXAP-08 wild-type strain but absent in the mutants of ∆*Fawc1* and ∆*Fawc2*, respectively. To confirm the functions of these two genes, the wild-type *Fawc1* and *Fawc2* connected with their native promoters, and terminators were transformed into ∆*Fawc1* and ∆*Fawc2* to obtain the complementation strains. As FaWC1 possesses both signal input and output domains, the LOV and zinc finger (ZnF) domains, respectively, the truncated versions of FaWC1, lacking either LOV or ZnF domains (Figure 2), were expressed in the ∆*Fawc1* mutant to explore the functions of these domains in light signaling and other life aspects of *F. asiaticum*. All the fungal strains used in this study and their genotypes are listed in Table 1.

### 3.3. The Marker Responses to Light Signal Are Mediated by WCC and Dependent on LOV but not ZnF Domain of FaWC1 in F. asiaticum

**Figure 3:**
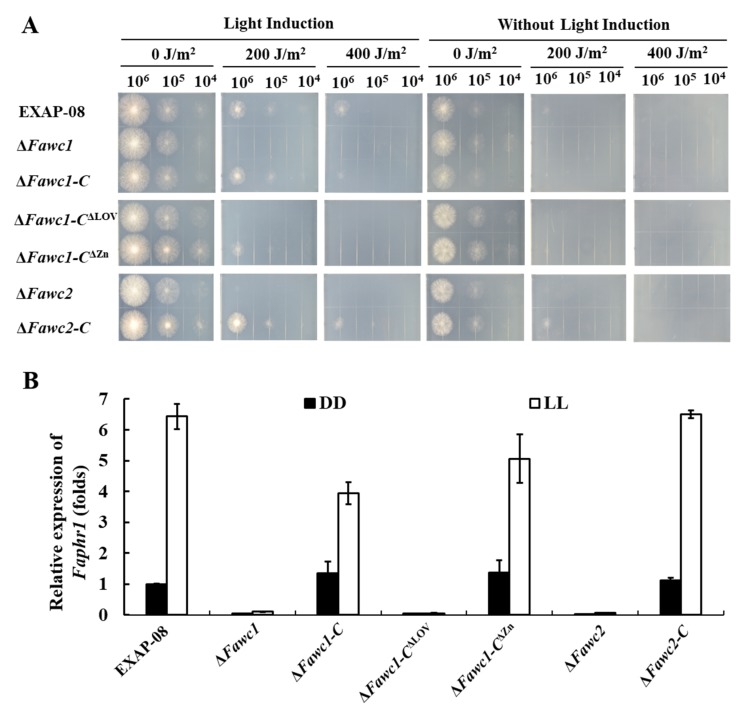
Effect of light on UV-C resistance. (**A**). Serial dilutions of all strains were point-inoculated onto complete agar medium (CM). After the UV irradiation of indicated dosages, the plates were incubated for one day in light (left) or darkness (right). (**B**). The relative expression level of the deduced photolyase gene *Faphr1* in wild-type and mutant strains as influenced by light. DD, samples cultured for 48 h in darkness; LL, samples experienced 47 h culture in darkness followed by one hour of light illumination. The bars present mean values ± SD of three replicate samples.

As fungi commonly sense light to indicate the presence of deleterious ultraviolet (UV) radiation, the light-induced survivability through UV damage is recognized as a marker response to light signal in fungi [35,36]. In the present study, the survival of serially diluted spores of all the tested strains was similarly poor if the fungal cultures were kept in the dark after UV-C treatment. However, when white light illumination was applied, the wild type (WT) could survive much better from UV-C irradiation than those cultures in the dark, but no recovery of survival spores was observed with ∆*Fawc1* and ∆*Fawc2*. The complementation strains, ∆*Fawc1-C* and ∆*Fawc2-C*, demonstrated similar photoreactivation levels as the WT, indicating that the defect in the light-induced UV-C resistance in the ∆*Fawc1* and ∆*Fawc2* mutants is indeed caused by the loss of these two genes (Figure 3A). Interestingly, the ∆*Fawc1-C*^∆LOV^ showed similar UV-C susceptibility as the ∆*Fawc1* and ∆*Fawc2* mutants, while in contrast, the ∆*Fawc1-C*^∆ZnF^ restored UV-C tolerance to the WT level. Gene expression analysis showed that the transcript level of the photolyase gene Faphr1 was significantly induced by light in WT, ∆*Fawc1-C*, ∆*Fawc2-C*, and ∆*Fawc1-C*^∆ZnF^ strains; however, no remarkable change of *Faphr1* expression as influenced with light has been observed in the ∆*Fawc1*, ∆*Fawc2*, and ∆*Fawc1-C*^∆LOV^ strains (Figure 3B).

**Figure 4:**
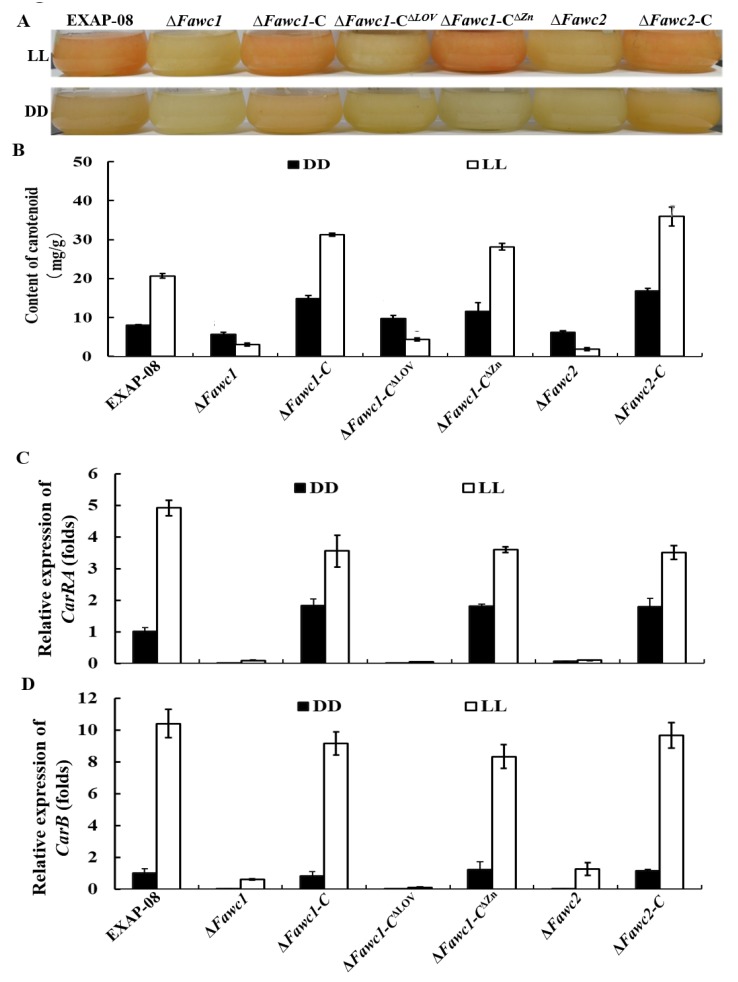
Effect of light on carotenogenesis. (**A**). Carotenoid pigment accumulation of the tested strains cultured in liquid CM for four days under constant light (LL) or darkness (DD). (**B**). Measurement of carotenoid contents in the mycelium of each strain harvested from the liquid shaking culture in (**A**). (**C**) and (**D**). Relative expression levels of deduced carotenoid biosynthesis genes *CarRA* and *CarB* in wild-type and mutant strains under light (LL) or darkness (DD). The bars in **B**, **C**, and **D** present mean values ± SD of three replicate samples.

Another marker response to the light signal in fungi is pigment production [18,37]. The WT strain could produce significantly more orange-colored carotenoid pigment in constant light compared to dark condition after growth in liquid complete medium (CM) for three days (Figure 4A). In contrast, there was no observable orange pigment accumulated by ∆*Fawc1* and ∆*Fawc2* in light and dark conditions (Figure 4A). Similarly, the ∆*Fawc1-C*^∆LOV^ mutant, which lacks the LOV domain of FaWC1, showed similar pigmentation phenotypes as the ∆*Fawc1* and ∆*Fawc2* mutants. However, the ∆*Fawc1-C*^∆ZnF^ with truncating the ZnF domain of FaWC1 demonstrated enhanced carotenogenesis in response to light, which was similar to the WT strain. Quantitative measurement assay also showed that in darkness, all strains produced basic carotenoid levels, and light treatment caused a significant increment of carotenoid accumulation in WT, ∆*Fawc1-C*, ∆*Fawc2-C*, and ∆*Fawc1-C*^∆ZnF^ strains, but light failed to alter the pigmentation behavior in ∆*Fawc1*, ∆*Fawc2-C*, and ∆*Fawc1-C*^∆LOV^ strains (Figure 4B). Gene expression analysis showed that the transcript levels of the carotenoid biosynthetic genes *CarRA* and *CarB* [37,38] were up-regulated in light versus dark condition in the WT, ∆Fawc1-C, ∆Fawc2-C, and ∆*Fawc1-C*^∆ZnF^ strains. Contrarily, the expression of *CarRA* and *CarB* could not be induced by light in the ∆*Fawc1*, ∆*Fawc2*, and ∆*Fawc1-C*^∆LOV^ strains (Figure 4C,D).

Collectively, the above data suggest that both FaWC1 and FaWC2 are responsible for light signaling to induce the marker responses, including carotenoid accumulation and UV damage tolerance. Moreover, the LOV and ZnF domains of FaWC1 are required and dispensable, respectively, for mediating the light responses in *F. asiaticum*.

### 3.4. Perithecia Maturation and Ascospore Development of F. asiaticum Are Regulated by WCC Photoreceptor

**Figure 5:**
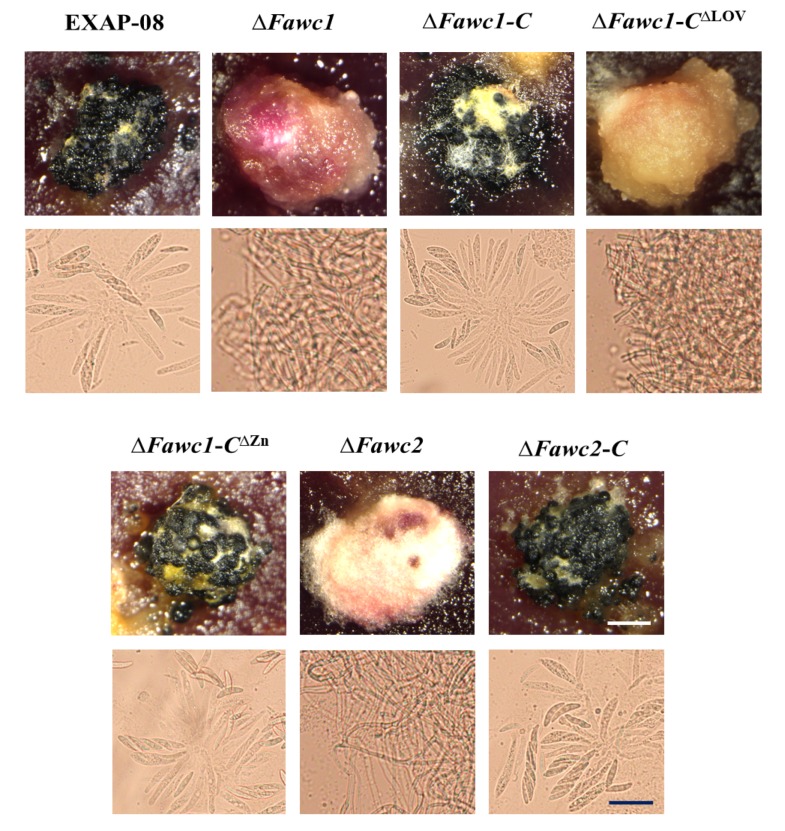
Regulation of *Fawc1* and *Fawc2* on the sexual reproduction development processes of *F. asiaticum*. The perithecium formed by each strain on carrot agar medium was observed via stereomicroscope. Via picking up the perithecia and pressing them on glass slides for microscopic analysis, mature ascospores were observed from the perithecia of EXAP-08, ∆*Fawc1-C*, ∆*Fawc1-C^∆^*^ZnF^, and ∆*Fawc2-C* strains, while in contrast, only mycelium biomass could be found in the immature perithecium of ∆*Fawc1*, ∆*Fawc2*, and ∆*Fawc1-C^∆^*^LOV^ mutant strains.

Sexual reproduction is usually vital for the dissemination of fungal pathogens in their lifecycles, and the near-UV light is known to induce the perithecia maturation and ascospore formation in FGSC [39]. However, two independent studies reported inconsistent effects of the WCC photoreceptor on the sexual development of *F. graminearum*, which was probably due to the difference of the wild-type background strains used in each study [30,40]. The present work with *F. asiaticum* showed that the WT strain was able to form mature perithecia, in which the sexual ascospores could be found apparently. In contrast, the ∆*Fawc1* and ∆*Fawc2* mutants could produce a comparable amount of perithecia as WT, but these mutants’ perithecia failed to develop into the black-pigmented mature stage, being deficient in ascospore formation (Figure 5). Re-introducing the wild-type *Fawc1* and *Fawc2* into the corresponding gene deletion mutants had fully recovered their perithecia maturation and ascospore formation abilities, suggesting that these WCC photo receptor components, FaWC1 and FaWC2, are indeed required for sexual reproduction development in this fungus. Additionally, the ∆*Fawc1-C^∆^*^ZnF^ and ∆*Fawc1-C^∆^*^LOV^ demonstrated phenotypes in sexual development as WT and ∆*Fawc1*, respectively, indicating that it should be the LOV domain, but not the ZnF domain, that is required for mediating the light signal to regulate the sexual reproduction of *F. asiaticum*.

### 3.5. FaWC1 and FaWC2 Play Different Roles in Regulating Virulence Expression

**Figure 6:**
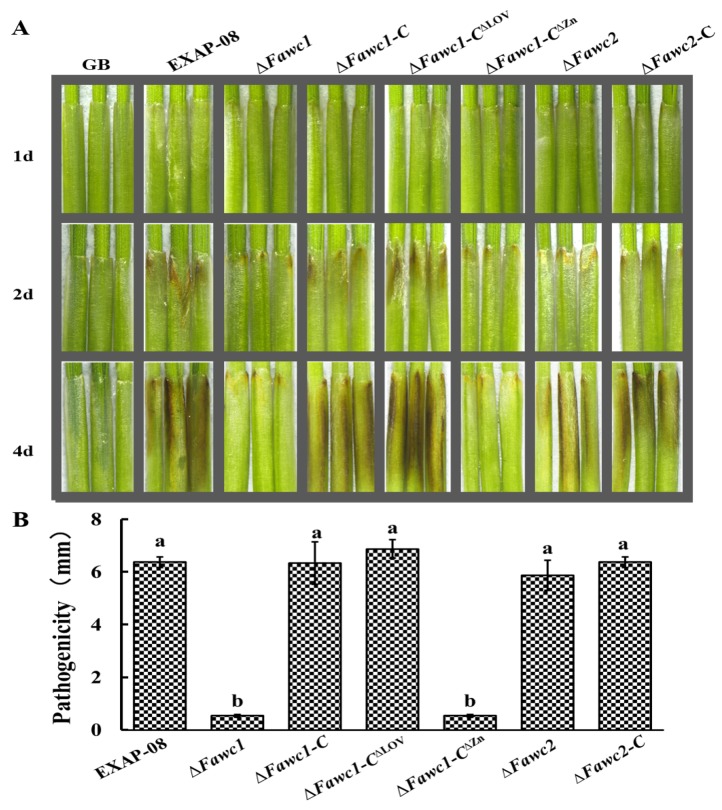
Virulence assay on wheat confirms that *Fawc1* regulate the virulence of *F. asiaticum* in a light-independent manner. (**A**). Wheat coleoptiles were inoculated with 5 µL conidial suspensions and were kept humid inside a plastic box. Fungal strains for test include the wild-type EXAP-08, ∆*Fawc1*, ∆*Fawc1*-*C*, ∆*Fawc1-C^∆^*^LOV^, ∆*Fawc1-C^∆^*^ZnF^, ∆*Fawc2*, and ∆*Fawc2*-*C*. Photographs were taken at four days post inoculation. (**B**). Statistical analysis of lesion sizes caused by each fungal genotype. Different letters represent a significant difference at *p* < 0.05. The bars present mean values ± SD (*n* = 20).

In the infection assay with wheat coleoptiles, inoculation with the WT caused apparent brown rot symptom in the host plant materials (Figure 6). In contrast, the ∆*Fawc1* mutant showed more than 80% reduction in pathogenicity in comparison with WT. Meanwhile, the complementation strain ∆*Fawc1-C* had a recovered pathogenicity level similar to that of the WT strain, suggesting that FaWC1 is involved in regulating the pathogenicity of *F. asiaticum*. However, the mutant with the deletion of *Fawc2* caused equivalent disease severity to the WT. These data suggested that FaWC1 and FaWC2 played independently different roles in regulating the pathogenicity of this fungus.

In order to assess whether and how light signaling via the WCC pathway could be involved in fungal pathogenicity expression, the mutant strains lacking the LOV and ZnF domains of FaWC1 were further analyzed in the host infection assay. Unexpectedly, the ∆*Fawc1-C*^∆LOV^ mutant showed similar pathogenicity as the WT and complementation strains. While in contrast, the pathogenicity of the ∆*Fawc1-C*^∆ZnF^ mutant was similar to the ∆*Fawc1* mutant, being significantly reduced compared to WT (Figure 6). Consequently, it can be concluded that the FaWC1 LOV domain, which is required for sensing light signals, exerts no influence on pathogenicity; on the other hand, the ZnF domain of FaWC1 is involved in regulating pathogenicity, although this domain is dispensable for mediating light signals in *F. asiaticum*.

## 4. Discussion

Light is a strong environment cue orchestrating numerous biological processes in various organisms. Understanding the photobiology mechanisms in fungi can be useful in designing feasible strategies to decrease the detrimental effects of fungi while enhancing those qualities that are beneficial. Most known fungal responses to light are mediated by the well-conserved fungal blue light receptor white collar complex (WCC) [41,42]. In this paper, we functionally investigated the WCC orthologs, *Fawc1* and *Fawc2*, in the plant pathogenic species *F. asiaticum*.

The FaWC1 and FaWC2 characterized in *F. asiaticum* strain EXAP-08 showed high similarity to their counterparts in *F. graminearum* sensu stricto, although they are distinct from each other in geographic, metabolic, and pathogenic phenotypes [9,16,43,44]. The FaWC1 contains a LOV domain for binding the flavin chromophore, a nuclear localization domain (NLS), a Zinc finger domain (ZnF) for DNA binding and transcription factor functions, and a PAS domain for protein–protein interaction. The FaWC2 also contains a PAS domain, NLS domain, and ZnF domain. All these protein domain characteristics of FaWC1 and FaWC2 are highly similar to WC-1 and WC-2 of *N. crassa*, respectively. Light generally increases cellular metabolism and at the same time causes significant oxidative stress to the organism, and to deal with this stress, protective pigments, e.g., carotenoids, are made [45]. Stimulated carotenogenesis as influenced with light treatment is a common photoresponsive phenotype among filamentous fungi [25], including several *Fusarium* species. Experiments in this study showed a clear dependence on WCC for light-induced carotenoid biosynthesis in *F. asiaticum*.

Another common reason for fungi to sense light should be to survive through the environmental stress posed by sunlight, especially the radiation of ultraviolet (UV), which can cause DNA damage or induce the accumulation of toxic reactive oxygen species (ROS) [45]. The ability to sense less harmful wavelengths of visible light can help the organisms to anticipate the emergence of, and promote tolerance to, the harmful UV. This premise has been shown among diverse fungal species through functional study with the orthologs of blue light receptor WCC [46]. It is the photoinduction of DNA repair enzymes, photolyases, and UV endonucleases, by the conserved blue light signaling pathway (WCC) that should be mainly responsible for the light-induced UV resistance in varied fungi [40,47,48,49,50]. In the present study, this reliable discipline has been confirmed in *F. asiaticum* in which the loss of *Fawc1* or *Fawc2* resulted in a deficiency of light-induced expression of photolyase gene (*Faphr1*) and consequently hypersensitivity to UV. Taken together, the two marker responses to light signals, including the expression of pigment synthesis and DNA repair enzymes to protect the fungal cells from the damage caused by UV during sunlight exposure, are indeed mediated cooperatively by FaWC1 and FaWC2 in *F. asiaticum*.

Fungal development can also be regulated by light signals. According to varied reports, light signals should have specific roles in certain species including *Aspergillus nidulans* [51], *Cercospora zeae-maydis* [27], *Botrytis cinerea* [29], and *Alternaria alternate* [52]. A key factor required in the regulation of fungal reproductive development events is the velvet complex composed of VeA, VelB, and LaeA [53], and the interactions between photoreceptors and velvet proteins have been demonstrated in *A. nidulans* [54]. As for the FGSC, the development of perithecia and production of ascospores are recognized to be important for the success of these pathogens in their disease cycle [55]. As found in the *F. graminearum* strain Z-3639, WCC should be required for the normal maturity of perithecia during sexual development [40]. However, another group later reported that WCC could negatively regulate sexual development in another strain of *F. graminearum*; the Z-3643 [30]. In the present study, the perithecial maturation and ascospore formation of *F. asiaticum* are dependent on the presence of the WCC. Consequently, the role of the conserved light receptors in regulating fungal reproductive development could be varied case by case.

Besides fungal development and metabolism, the involvement of light receptors in fungal pathogenesis is increasingly attracting research attention [20]. The orthologs of WCC have been recognized in several pathogenic species. However, functional differences have also been reported among them [27,28,29,30,40,52]. For example, in *Magnaporthe oryzae*, constant light suppresses disease development, which is mediated via MGWC-1 [28]. In the maize leaf pathogen *C. zeae-maydis*, the WC-1 ortholog Crp-1 is required for stomata tropism and for appressorium and lesion formation, implying that Crp-1 positively contributed to its virulence [27]. While in the necrotrophic pathogen *B. cinerea*, the WCC and its direct target BcLTF1, a light-responsive transcription factor homologous to the SUB1 in *N. crassa*, are required to cope with the oxidative stress that is caused by either excessive exposure to light or arising during host invasion, and thus required for achieving full virulence under excessive light [29].The present study with *F. asiaticum* shows that the deletion of *Fawc1* resulted in decreased virulence. This is in contrast with the reports of *F. graminearum*, in which the WCC seems to be not involved in regulating virulence [30,40]. Even in those fungal species in which WCC are involved in determining virulence expression, the ways are quite different from each other; for example, WCC orthologs are involved in affecting the infection activities of *B. cinerea*, *C. zeae-maydis*, and *M. oryzae* in a light-dependent manner [27,28,29]. While in contrast, WCC are required for the full virulence of *C. neoformans* and *F. oxysporum* in mammals in a light-independent way [20,47,49]. Since the WCC proteins possess domains for both signal input (LOV domain) and output (Zn-finger domain), it occurs in several fungi that the WCC orthologs have both light-dependent and independent regulatory roles; for example, in *Trichoderma reesei*, the WCC photoreceptors, BLR1 and BLR2, play a role in the alteration of carbohydrate metabolic functions and the transport of compounds with distinct, both positive and negative targets in darkness [25].

To address the issue that whether the light signal and light-perceiving ability by fungi can affect pathogenicity, we have further revealed that the LOV domain of FaWC1 is required, while the ZnF domain is dispensable for inducing carotenoid synthesis and UV tolerance. These findings imply that the WCC-mediated light signaling in *F. asiaticum* does rely on the LOV domain for perceiving light signals, while the ZnF domain of FaWC1 seems to be not involved in regulating the downstream light responses. This phenomenon can be interpreted according to what has been found in several other fungi that indicates that the WC-2 ZnF domain is indeed responsible for the light signal output to regulate the expression of downstream genes [56]. More importantly, the ZnF domain of FaWC1 should have its downstream targets, which are not for light signaling but most likely for pathogenicity regulation. These findings have raised two open questions for future study: (1) to identify the light-independent targets of FaWC1 that are involved in pathogenicity expression; and (2) to resolve the upstream mechanisms that can orchestrate the photoreceptor to well balance between sensing light and regulating light-independent pathogenicity behaviors.

## 5. Conclusions

In the present study with *F. asiaticum*, the conserved WCC photoreceptor orthologs, namely FaWC1 and FaWC2, have been characterized. Both FaWC1 and FaWC2 are required for perceiving light signals to regulate UV resistance, secondary metabolism, and sexual reproduction. However, FaWC1 and FaWC2 performed diverged roles in virulence expression. Based on the genetics data, we conclude that the WCC orthologs in *F. asiaticum* are responsible for light signaling to adapt to the environmental niche; meanwhile, moonlighting functions of the individual component FaWC1 are required for regulating pathogenicity in a light-independent manner.

## Figures and Tables

**Table 1:** *F. asiaticum* strains used in this study. LOV: Light–oxygen–voltage, ZnF: Zinc finger.

Name	Genotype	Reference
EXAP-08	Wild type	[10]
∆*Fawc1*	Knockout mutant, *Fawc1*::*Hyg*	This study
∆*Fawc2*	Knockout mutant, *Fawc2*::*Hyg*	This study
∆*Fawc1-C*	*Fawc-1* complemented transformant of Δ*Fawc-1*	This study
∆*Fawc2-C*	*Fawc-2* complemented transformant of Δ*Fawc-2*	This study
∆*Fawc1-C*^∆LOV^	LOV domain deletion mutant	This study
∆*Fawc1-C*^∆ZnF^	ZnF domain deletion mutant	This study

## Supplementary Materials

The following are available online at https://www.mdpi.com/2076-2607/8/3/365/s1, Table S1: All the primers used in the mutant generating and diagnosis PCR reactions.
