# Supplementary material for: The Photoreceptor Components FaWC1 and FaWC2 of Fusarium asiaticum Cooperatively Regulate Light Responses but Play Independent Roles in Virulence Expression
Source: Microorganisms. 2020 Mar 5;8(3):365. doi: 10.3390/microorganisms8030365 (PMC7143034; doi:10.3390/microorganisms8030365)
Supplement: Supplementary file 1 [file microorganisms-08-00365-s001.pdf]

Supplementary Table S1. All the primers used in the present study.

Primer name	5'-3'	Target of Amplification
Q *β-tubulin*-F	ACGGCACGGGGAACATACTTG	qRT-PCR of *β-tubulin*
Q *β-tubulin*-R	TCGAGCGCATGAGCGTTTACT	
Q *CarRA*-F	GTTGCTTCCGGTCAAGAAATG	qRT-PCR of *CarRA*
Q *CarRA*-R	CCAGGACAGAGGAAGGTAGA	
Q *CarB*-F	GGAGTCAAACGACAGCATCTT	qRT-PCR of *Car B*
Q *CarB*-R	AGTACAACAACCGCATCCTTAC	
Q *Phr1*-R	GGACCGGCCTTTCGATTATAC	qRT-PCR of *Phr1*
Q *Phr1*-F	GAGTGAGAAGTTTGACCCTGAC	
Fawc2-U-F (P7)	CTAAAGACAGGACAGGGACTC	Generation of knock-out cassette of *Fawc2*
Fawc2-U-R (P11)	CCACAGCTGCAGTCTAGAGCGGTTATCAAGGTTACGTTTGC	
Fawc2-Hph-F (F9)	GCAAACGTAACCTTGATAACCGCTCTAGACTGCAGCTGTGG	
Fawc2-Hph-R (F10)	CACATTTGCGACTGTCGAGTCGGGATCCGCTTAGACAAC	
Fawc2-D-F (P12)	GTTGTCTAAGCGGATCCCGACTCGACAGTCGCAAATGTG	
Fawc2-D-R (P8)	GGGGTAATCAAAGCTCTCCT	
Hph-F (P5)	CGGCGTAGGGTTGTTCC	Identification of hygromycin in the Δ*Fawc1* or Δ*Fawc2*
Hph-R (P6)	TGGCGACCTCGTATTGG	
Fawc2-F (P3)	ACTTTTATCCGCTTTTCT	Identification target segment of *Fawc2*
Fawc2-R (P4)	GTTGTTGTTGACCTCCAC	
Com-Fawc2-F	CGATATCTCTAGAGGATCCTGGGTTTCCTTGGTTGAGAT	Construction of *Fawc2* complemented segment
Com-Fawc2-R	TGTCTACTGCTGGCCTAGTACTGTCCTGCTGTGGTTCG	
G418-F	GGAAGCGGTCAGCCCATTCG	Identification of Geneticin in the Δ*Fawc1-C* or Δ*Fawc2-C*
G418-R	CCGTGTTCCGGCTGTCAGCG	
Fawc1-U-F (P7)	CTTGTGCCAAGTTGCCAGCAATC	Generation of knock-out cassette of *Fawc1*
Fawc1-U-R (P11)	CCACAGCTGCAGTCTAGAGCGGCGACAGCATCTGGCTGGAA	
Fawc1-Hph-F (F9)	TTCCAGCCAGATGCTGTCGCCGCTCTAGACTGCAGCTGTGG	
Fawc1-Hph-R (F10)	ATGAATCACGCCCAACGCCGGGATCCGCTTAGACAAC	
Fawc1-D-F (P12)	GTTGTCTAAGCGGATCCCGGCGTTGGGCGTGATTCAT	
Fawc1-D-R (P8)	GGAAGCCACTGACGTCAATC	
Fawc1-F (P3)	TGCCTCTGTTCCTCCTGA	Identification target segment of *Fawc1*

Fawc1-R (P4)	CACGGATGAGGACAGTTAAG	
Com-Fawc1-F	GATCCTCTAGGTACCCGGGGATCCCTCAAGTCCCTAGGTTTCCC	Construction of *Fawc1* complemented segment
Com-Fawc1-R	GGCCATCTCGAGCGGGATCCGAGATTGGCTTGTCTCGCG	
Fawc1-C$^{\Delta LOV}$-F	TAGTTGCTACGCGCAAAAACGGTGCCGTGTATAACCTGAA	Generation of knock-out LOV domain in the *Fawc1*
Fawc1-C$^{\Delta LOV}$-R	TTCAGGTTATACACGGCACCGTTTTTGCGCGTAGCAACTA	
Fawc1-C$^{\Delta Zn}$-F	GCGTAAGAGAAGAAAGGGAGTCGGCGATTCTCGAAGCAAGAAGTC	Generation of knock-out ZnF domain in the *Fawc1*
Fawc1-C$^{\Delta Zn}$-R	GACTTCTTGCTTCGAGAATCGCCGACTCCCTTTCTTCTCTTACGC	
Fawc1-F	ATGGATGGCTTCTACTCC	Amplify *Fawc1* fragment in the *Fawc1-C*$^{\Delta LOV}$ or *Fawc1-C*$^{\Delta Zn}$ for sequencing
Fawc1-R	TCAAGATTGGCTTGTCTC	
